# Perturbation of host ubiquitin systems by plant pathogen/pest effector proteins

**DOI:** 10.1111/cmi.12385

**Published:** 2014-11-25

**Authors:** Mark J Banfield

**Affiliations:** Department of Biological Chemistry, John Innes CentreNorwich Research Park, Norwich, NR4 7UH, UK

## Abstract

Microbial pathogens and pests of animals and plants secrete effector proteins into host cells, altering cellular physiology to the benefit of the invading parasite. Research in the past decade has delivered significant new insights into the molecular mechanisms of how these effector proteins function, with a particular focus on modulation of host immunity-related pathways. One host system that has emerged as a common target of effectors is the ubiquitination system in which substrate proteins are post-translationally modified by covalent conjugation with the small protein ubiquitin. This modification, typically via isopeptide bond formation through a lysine side chain of ubiquitin, can result in target degradation, relocalization, altered activity or affect protein–protein interactions. In this review, I focus primarily on how effector proteins from bacterial and filamentous pathogens of plants and pests perturb host ubiquitination pathways that ultimately include the 26S proteasome. The activities of these effectors, in how they affect ubiquitin pathways in plants, reveal how pathogens have evolved to identify and exploit weaknesses in this system that deliver increased pathogen fitness.

## Introduction

Post-translational modification is a tool used by prokaryotic and eukaryotic cells to regulate protein function. These modifications enable diverse outcomes on target proteins. Addition/removal of small molecules [e.g. phosphate (phosphorylation), acetate (acetylation) and sulphate (sulphation)] can directly regulate activity or promote protein/protein interactions. Addition of larger functional groups [e.g. hydrophobic groups (myristoylation/palmitoylation) or sugars (glycosylation)] can define protein localization to a membrane or enhance stability. Post-translational modification also includes structural changes such as the formation of intramolecular disulphide or isopeptide bonds that promote protein stability.

Attachment of other polypeptides, such as ubiquitin and the structurally related but sequence-diverse ubiquitin-like proteins (e.g. SUMO, NEDD8), to substrate proteins modulates many biological processes from the cell cycle and cell division to apoptosis and the immune response and inflammation (Pickart, 2001; Kerscher *et al*., 2006; Rotin and Kumar, 2009). Ubiquitination of target proteins is a tightly regulated process controlled by a three-step enzyme cascade involving activating (E1), conjugating (E2) and ligating (E3) reactions (Berndsen and Wolberger, 2014). The E3 ubiquitin ligases are responsible for specificity as they define the substrates of ubiquitination. Ubiquitin can be ligated to target proteins via different lysine residues and as a monomer or a polyubiquitin chain, occasionally ligation occurs via the amino group of the N-terminal methionine (Ciechanover and Ben-Saadon, 2004). The best understood ubiquitin modification is via Lys48, which targets substrate proteins for degradation via the 26S proteasome. Ubiquitination can be reversed by the action of specific deubiquitination enzymes (Reyes-Turcu *et al*., 2009), recycling ubiquitin and competing with E3 ligases.

Adapted pathogens and pests of plants suppress host cell defences by delivering effector proteins into host cells (Dodds and Rathjen, 2010; Dou and Zhou, 2012; Petre and Kamoun, 2014). Bacterial pathogens use type III or type IV secretion systems to inject effector proteins into cells, while delivery of translocated filamentous pathogen effectors most likely occurs via haustoria (Dodds and Rathjen, 2010; Petre and Kamoun, 2014) or other specialized structures such as the biotrophic interfacial complex (BIC; Giraldo *et al*., 2013). Effectors can also be secreted in the saliva of nematodes and insects (Hogenhout *et al*., 2013). The ubiquitination system has emerged as a particular focus of effector protein activity during pathogenesis in both animal and plant cells (Spallek *et al*., 2009; Trujillo and Shirasu, 2010; Marino *et al*., 2012; Ashida *et al*., 2014).

**Fig 1:**
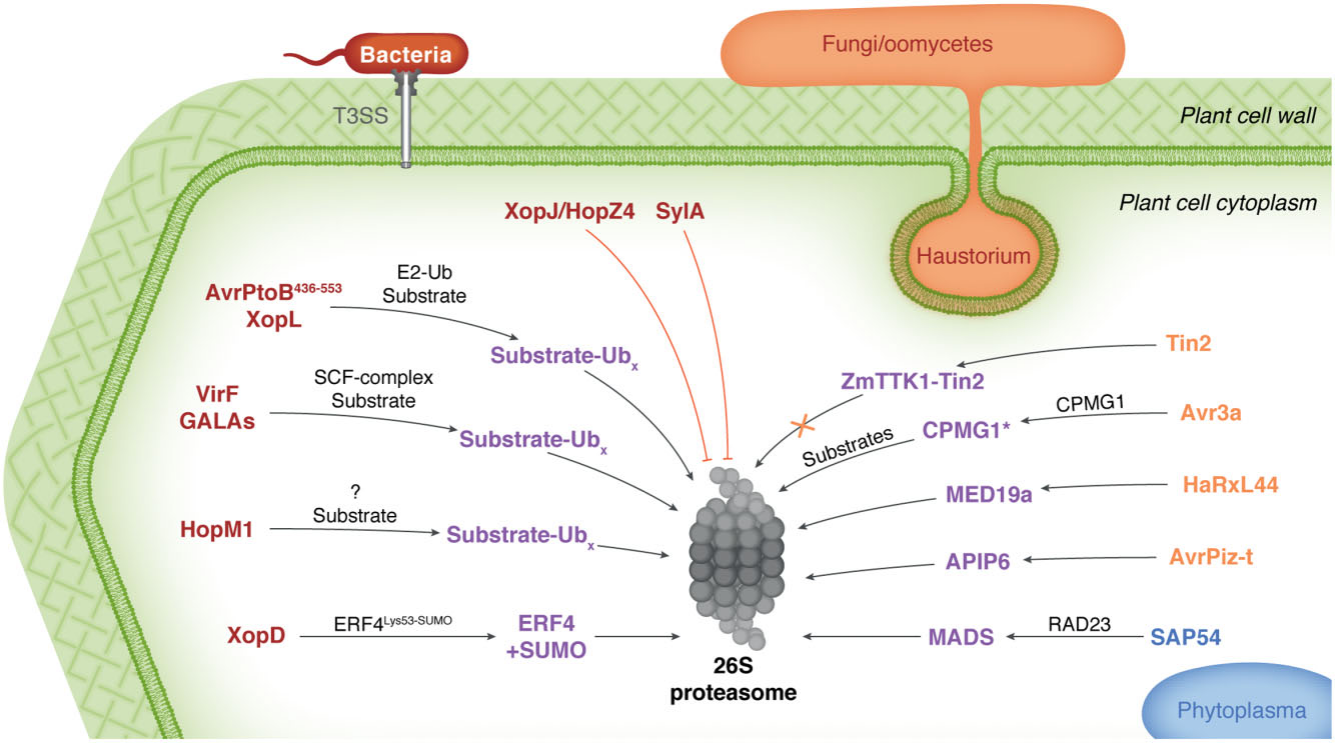
Overview of plant pathogen effectors that perturb host ubiquitin systems and their activities. Bacterial effectors secreted through the type III secretion system are shown in beige, the phytoplasma effector is shown in blue and fungal/oomycete effectors are shown in brown.

In this review, I detail selected examples (Fig. 1) of how prokaryotic and eukaryotic pathogens and pests of plants use effector proteins to perturb the host ubiquitin system for their own benefit to promote colonization, providing updates to previous reviews on the subject where possible [viral proteins will not be covered here, but have been previously (Marino *et al*., 2012)]. Readers are referred to other articles in this themed issue, and other excellent recent reviews [e.g. (Ashida *et al*., 2014)], for examples of how pathogens of animals use effector proteins to manipulate host ubiquitin systems.

## Well-established systems: bacterial effectors

Effector proteins of bacteria that are translocated into host cells by the type III and type IV secretion system have been the subjects of intensive study. A significant subset of these proteins have been observed to interact with components of the host ubiquitin-proteasome system (UPS). In addition, proteins with a standard secretion signal and natural products can also perturb the host UPS.

### E3 ligases

*Pseudomonas syringae* pv. *tomato* DC3000 is the causative agent of bacterial speck disease on tomato and *Arabidopsis*. The genome of *P. syringae* DC3000 encodes at least 28 type III secreted effector proteins (Xin and He, 2013). One of these effectors, AvrPtoB, is a multi-domain protein that contains two ordered helical bundle regions (residues 121–205 and 250–359) that interact with the intracellular kinase domain of the plasma membrane receptor-like kinases FLS2 (Gohre *et al*., 2008), CERK1 (Gimenez-Ibanez *et al*., 2009), Bti9 (Zeng *et al*., 2012) and BAK1 (Cheng *et al*., 2011) and cytoplasmic kinases Pto (Dong *et al*., 2009) and Fen (Rosebrock *et al*., 2007), to inhibit immunity-related signalling (reviewed in Wirthmueller *et al*., 2013). Protein structure analysis revealed that the third ordered domain to the C-terminus of the protein (residues 436–553) is a mimic of eukaryotic U-box and RING-finger E3 ligases (Abramovitch *et al*., 2006; Janjusevic *et al*., 2006). AvrPtoB was shown to interact with eukaryotic E2 conjugation enzymes and ubiquitin and to catalyse auto-ubiquitination (Abramovitch *et al*., 2006; Janjusevic *et al*., 2006), demonstrating E3 ligase activity. The first identified target of AvrPtoB E3 ligase activity *in trans* was the tomato immunity-related kinase Fen (Rosebrock *et al*., 2007). Fen, a Pto homologue, is ubiquitinated in the presence of AvrPtoB *in vitro* using recombinant proteins. In tomato protoplasts, AvrPtoB promotes 26S-proteasome-dependent degradation of Fen. Interestingly, unlike Fen, the immunity-related kinase Pto escapes ubiquitination by AvrPtoB, possibly by phosphorylating residue Thr450 in the E3 ligase domain of this effector (Ntoukakis *et al*., 2009). However, a recent study suggests that the Pto kinase activity is not required for the escape of ubiquitination. Instead, Pto can bind to both the helical bundle regions of AvrPtoB, one proximal to the E3 ligase domain (residues 250–359, which leads to Pto ubiquitination) and one distal (residues 121–205). Pto binding to the AvrPtoB distal region escapes ubiquitination (Mathieu *et al*., 2014). The E3 ligase activity of AvrPtoB has also been shown to target the intracellular kinase domains of plant pattern recognition receptors such as FLS2 and EFR *in vitro* and promote degradation of FLS2 *in planta* (Gohre *et al*., 2008). In both plants and mammals, it is well established that the specificity of protein substrates destined for ubiquitination is dictated by E3 ligases (Deshaies and Joazeiro, 2009), with over 600 RING-E3 ligases are encoded in mammalian genomes (Li *et al*., 2008). The function of these E3 ligases can be modified by post-translational modifications and binding partners (Deshaies and Joazeiro, 2009). For AvrPtoB, it appears that the N-terminal helical bundle domains may act as specificity determinants, orientating immunity-related kinases or receptor-like kinases such that the AvrPtoB E3 ligase domain can catalyse their ubiquitination. Finally, while the E3 ligase activity of AvrPtoB is likely important for regulation of immunity-related kinases, it may not always be strictly necessary for inactivation of these enzymes. In certain cases, the N-terminal AvrPtoB helical bundle domains alone may be sufficient to interfere with kinase signalling (Zeng *et al*., 2012).

*Xanothomonas campestris* pv. *vesicatoria* causes bacterial leaf spot on pepper and tomato. Recently, a novel type III secreted effector from this pathogen was shown to exhibit E3 ligase activity. XopL interacts with specific E2 conjugating enzymes (including two from *Arabidopsis*) and predominantly catalyses Lys11-linked polyubiquitin chains *in vitro* using recombinant proteins (Singer *et al*., 2013). The E3 ligase activity was located to the C-terminal part of the effector, encompassing residues 474–660, and this region of the protein was important for provoking programmed cell death. Interestingly, unlike the C-terminal domain of AvrPtoB, the crystal structure of the E3 ligase region of XopL is novel and does not share any homology with E3 ligases whose structure is known. Further, XopL_474–660_ lacks any Cys residues, suggesting the ligase activity does not proceed via a thioester intermediate. The molecular mechanism of ligation currently remains unknown, as do the cellular targets of XopL involved in suppressing plant defence. It is possible that the N-terminal leucine-rich repeat region of XopL mediates substrate selection through protein/protein interactions.

### F-box proteins

*Agrobacterium tumefaciens* causes crown gall disease in susceptible plants. Infection requires the transfer of a small segment of DNA (the T-DNA), through a type IV secretion system, from a pathogen-encoded virulence plasmid into the plant genome. This activity is widely used for plant transformation with heterologous genes. One gene encoded on the virulence plasmid is VirF, an F-box motif-containing protein that interacts with *A. tumefaciens* VIP1 and VipE2 and targets them for degradation in the plant cell nucleus via a host SCF (Skp1-Cdc53-cullin-F-box) complex and the 26S proteasome (Tzfira *et al*., 2004). This targeted degradation frees the T-DNA, which was bound by multiple VipE2 molecules, allowing integration into the plant genome. Interestingly, VirF is not required for infection of some plants and it is thought that host F-box proteins can functionally complement VirF function (Zaltsman *et al*., 2010). Further, other host-encoded F-box proteins may also be involved in destabilization of proteins during *A. tumefaciens* transformation via polyubiquitination (Anand *et al*., 2012).

*Ralstonia solanacearum* causes bacterial wilt in a range of important crop plants including potato, tomato, banana and pepper. Among the suite of type III effectors encoded in the *R. solanacearum* genome are the GALA proteins (named after a GAxALA motif in their sequence; Angot *et al*., 2006; Remigi *et al*., 2011). In addition to the GAxALA motif, GALA proteins also encode an F-box domain, which was shown to be essential for GALA7's virulence function during infection of *Medicago truncatula* (Angot *et al*., 2006). It is hypothesized that GALAs interfere with ubiquitin-mediated protein degradation by reconstituting host SCF complexes to promote disease. However, confirmation of this requires that the targets of GALA-mediated ubiquitination are identified and their roles in infection characterized.

### SUMO de-conjugation

The *X. campestris* pv. *vesicatoria* genome encodes XopD, a type III secreted effector that specifically cleaves the ubiquitin-like molecule SUMO following an invariant di-Gly motif towards the C-terminus and de-conjugates SUMO from targeted substrate proteins (Hotson *et al*., 2003; Chosed *et al*., 2007). The SUMO-protease activity of XopD resides in its C-terminal domain, with the N-terminal region of the effector encoding a non-specific DNA-binding domain and EAR motifs. This suggested that XopD might interfere with host DNA transcription during pathogen infection (Kim *et al*., 2008). Recently, XopD has been shown to repress production of the plant hormone ethylene, and suppress ethylene-stimulated defence, by de-sumoylating the tomato transcription factor ERF4 at position Lys53 (Kim *et al*., 2013). De-sumoylation leads to destabilization of ERF4, dependent on the 26S proteasome, and represses transcription of both ethylene biosynthesis genes and ethylene-dependent reporter constructs in plant cells. Silencing of ERF4 in plants made them more susceptible to infection with *X. campestris* pv. *vesicatoria*, confirming a role for this protein in plant immunity.

### Interfering with vesicle trafficking

The *P. syringae* type III secreted effector HopM1 is one of a pair of functionally redundant genes (the second being avrE) that when deleted cause a severe virulence defect (DebRoy *et al*., 2004). Delivery of HopM1 targets the immunity-related Arabidopsis protein MIN7 (HopM1-interacting protein 7) for degradation by the 26S proteasome by causing its poly-ubiquitination, probably by acting as an adaptor protein (Nomura *et al*., 2006). *At*MIN7 is an ARF-GEF (adenosine diphosphate ribosylation factor, guanine nucleotide exchange factor) protein and HopM1 activity is specific for this particular ARF-GEF family member. The targeted degradation of *At*MIN7 interferes with vesicle trafficking and the deposition of callose, the latter being a well-characterized read-out of surface-mediated plant immunity pathways. *At*MIN7 is also involved in intracellular-mediated plant immunity pathways (Nomura *et al*., 2011). Recently, HopM1 expression in plant cells has revealed the suppression of reactive oxygen species burst and stomatal immunity in an *At*MIN7-independent fashion (Lozano-Duran *et al*., 2014). The effects of HopM1 were still 26S proteasome-dependent, but are mediated by interfering with the activity of the 14-3-3 protein GRF8 (*At*MIN10). Additional virulence associated targets of HopM1 activity have also been suggested (Gangadharan *et al*., 2013).

### Inhibition of proteasome activity

*Xanothomonas campestris* pv. *vesicatoria* effector XopJ is a member of the widespread YopJ family of cysteine proteases/acetyltransferases found in pathogens of plants and animals (Lewis *et al*., 2011). XopJ interacts with RPT6, a subunit of the 19S regulatory particle and reduces the activity of the complete 26S proteasome (Ustun *et al*., 2013). This activity is dependent on intact catalytic and N-myristoylation sites, although the molecular mechanism of inhibition is yet to be defined. XopJ also represses salicylic acid-mediated plant defence responses. XopJ's activities are hypothesized to enhance nutrient availability by prolonging host cell viability, increasing infection potential (Ustun *et al*., 2013). Further, *P. syringae* pv. *lachrymans* produces HopZ4, a close homologue of XopJ, that also interacts with RPT6 to inhibit the 26S proteasome during infection (Ustun *et al*., 2014). The activity of HopZ4 is functionally redundant with XopJ as it complements the loss of XopJ in *Xanthomonas* spp..

*Pseudomonas syringae* pv. *syringae* can infect many plant species but is best known for causing brown spot disease of bean. This pathogen produces a small natural product called SylA, via a non-ribosomal peptide/polyketide synthase route, which specifically binds to and inhibits the eukaryotic 26S proteasome (Groll *et al*., 2008). Targeted gene disruption, to prevent the synthesis of SylA, resulted in a pathogen with significantly attenuated virulence, suggesting that inhibiting the 26S proteasome can be necessary for full virulence. How the activity of SylA promotes pathogenesis of *P. syringae* remains unknown.

As detailed above, certain strains of plant pathogenic bacteria have evolved effectors to target host proteins to the proteasome for degradation (requiring a functional proteasome), but also inhibit proteasome activity. These activities appear to be antagonistic. However, during infection, the action of these effectors may be spatially or temporally separated. XopJ and HopZ4 are localized to the plant cell plasma membrane and may only target a subset of the total proteasome complexes in the cell (Ustun *et al*., 2013; 2014[Bibr b60],[Bibr b61]). Also, it is known from animal pathogens that type III secreted effectors can be delivered in a pre-established order (Lara-Tejero *et al*., 2011). Therefore, particular effectors may only be present in the host cell at certain times, and these may be mutually exclusive. It is also possible that when delivered together, effectors are able to fine-tune the activity of the proteasome and a balance in effector activities may provide maximal benefit to the pathogen.

### Promoting transcription factor degradation

Phytoplasmas are bacteria whose life cycle includes stages in both plants and insects, during which they secrete effector proteins (Sugio and Hogenhout, 2012). They can cause yield losses in crops as they interfere with plant developmental processes. Two phytoplasma effectors have been shown to induce destabilization of host transcription factors (Sugio *et al*., 2011; MacLean *et al*., 2014). One of these, SAP54, promotes degradation of MADS-box transcription factors, dependent on the UPS (MacLean *et al*., 2014). SAP54 does this by interacting with RAD23, a plant protein that shuttles substrates to the 26S proteasome. Intriguingly, this targeted degradation results in sterile plants displaying phyllody (conversion of flowers into leaves) that are then more attractive to insects, ultimately promoting phytoplasma dissemination.

## Emerging systems 1: eukaryotic pathogen effectors

The molecular mechanisms underlying the activity of effectors from eukaryotic pathogens of plants, including fungi and oomycetes, are less well characterized than those from bacteria. However, it is emerging that effectors from these parasites also target the host UPS to promote virulence.

### Stabilizing a U-box protein

The oomycete *Phytophthora infestans* was responsible for the Irish potato famine and remains an agriculturally relevant pathogen today as the causative agent of potato and tomato late blight. The most studied effector protein from *P. infestans* to date is the RXLR-type effector AVR3a (Armstrong *et al*., 2005; Whisson *et al*., 2007; Bos *et al*., 2009; 2010[Bibr b7],[Bibr b8]; Gilroy *et al*., 2011; Segretin *et al*., 2014). AVR3a has been shown to be important for virulence and to interact with and stabilize the immunity-related U-box E3 ligase protein CMPG1 (Bos *et al*., 2010). In the absence of AVR3a, CMPG1 is most likely degraded by the 26S proteasome as inhibitors of this macromolecular machine increase the abundance of this protein (Bos *et al*., 2010). AVR3a suppresses cell death induced by INF1 (an elicitin from *Phytophthora*) and other immunity-related recognition events at the plasma membrane, and these all require CMPG1 (Bos *et al*., 2010; Gilroy *et al*., 2011). It is hypothesized that stabilization of CMPG1 by AVR3a, by preventing its degradation by the proteasome, modifies its activity to interfere with immunity-related signalling.

### Degrading a component of the Mediator complex

*Hyaloperonospora arabidopsidis* is an oomycete pathogen of Arabidopsis and a model system for the study of oomycete/host interactions. The RXL effector protein HaRxL44 interacts with and specifically promotes the degradation of the Mediator subunit 19a, in a 26S proteasome-dependent manner, potentially acting as an adaptor protein (Caillaud *et al*., 2013). In yeast-2-hybrid assays, HaRxL44 also interacts with E3 ligases, as well as Med19a (Mukhtar *et al*., 2011), supporting this hypothesis. The mediator complex mediates interactions between transcriptional regulators and RNA polymerase II and maybe a target for a number of *H. arabidopsidis* effectors (Mukhtar *et al*., 2011). It is predicted that degradation of Med19a by HaRxL44 perturbs the balance in plant defence hormone signalling, through changes in gene expression, to enhance susceptibility to biotrophic pathogens.

### Masking of an ubiquitin-proteasome degradation motif

The pathogenic fungus *Ustilago maydis* causes corn smut disease in maize. During infection, *U. maydis* produces an effector protein called Tin2 (Tanaka *et al*., 2011). Tin2 masks a phosphodegron motif (Asp-Ser-Gly-X-Ser) present in a serine/threonine kinase termed ZmTTK1 (Tanaka *et al*., 2011), which promotes stability of the kinase in a 26S proteasome-dependent manner. The masking of the phosphodegron sequence by Tin2 produces a similar effect to phospho-dead mutations in this motif that also promote stability. The stabilization of ZmTTK1 by Tin2 is hypothesized to result in a change in metabolic flux, promoting anthocyanin rather than lignin biosynthesis. The reduction in lignin biosynthesis may prevent fortification of plant cell walls that would otherwise limit fungal access to plant tissue, providing a link from effector function to pathogen virulence.

### Suppressing the activity of an E3 ligase

The rice blast fungus *Magnaporthe oryzae* has a major impact on production of rice worldwide. Effector proteins of this fungus that are destined for translocation into the plant cell accumulate in a structure called the BIC (Giraldo *et al*., 2013). Expression of one *M. oryzae* effector, AvrPiz-t, in rice plants suppresses a variety of innate immune responses associated with early perception of the pathogen and enhances susceptibility (Park *et al*., 2012). AvrPiz-t interacts with an E3 ligase called APIP6 and suppresses its activity *in vitro*. Interestingly, APIP6 ubiquitinates AvrPiz-t *in vitro* and in plant cells. Co-expression of AvrPiz-t and APIP6 in plant cells leads to degradation of APIP6 when compared with APIP6 expression alone, suggesting that AvrPiz-t promotes degradation of APIP6 and this is dependent on the 26S proteasome (Park *et al*., 2012). Finally, APIP6 was shown to have a role in innate immunity of rice to *M. oryzae*, confirming APIP6 as a logical target for a pathogen effector and a causal link between AvrPiz-t translocation and enhanced susceptibility (Park *et al*., 2012).

## Emerging systems 2: nematodes/insect pest effectors

In addition to the prokaryotic and eukaryotic pathogens described above, it is increasingly recognized that other pests of plants, such as nematodes and insects, secrete effector proteins during infection to modulate the host innate immune system (Hewezi and Baum, 2013; Rodriguez and Bos, 2013).

*Globodera rostochiensis* is a nematode that infests potatoes and tomatoes. It expresses UBCEPs (ubiquitin carboxyl extension proteins) in gland cells and these proteins contain a signal for secretion (Chronis *et al*., 2013). When expressed in plant cells one UBCEP, *Gr*UBCEP12, is processed into free ubiquitin and the C-terminal extension resulting in the suppression of innate immunity. In *Gr*UBCEP12 overexpression lines, a component of the 26S proteasome was reduced in expression suggesting a role for the putative effector in interfering with proteasome function to promote infestation.

To date, there is yet to be an effector from an insect pest that is known to directly interfere with the host UPS. However, a putative ubiquitin-specific protease has been observed in an enriched Expressed Sequence Tag (EST) library from the salivary glands of the pea aphid *Acyrthosiphon pisum* (Carolan *et al*., 2011). This indicates there may be effectors secreted in the saliva of insects that perturb the host UPS during infection.

## Conclusion

The host UPS is a target of multiple plant pathogen and pest effector proteins, suggesting it has a fundamental evolutionarily conserved role in pathogenesis and/or immunity and is an ‘Achilles heel’ of the host. It would not be surprising if novel perturbations of the UPS by pathogens and pests of plants continue to be discovered. It is important to understand the molecular mechanisms of these activities as they may present routes for the design of novel strategies to engineer increased plant resistance to pathogens and pests of relevance to agriculture.
